# Surgical management of craniospinal axis malignant peripheral nerve sheath tumors: a single-institution experience and literature review

**DOI:** 10.1186/s12957-023-03227-y

**Published:** 2023-10-26

**Authors:** Ajmain Chowdhury, Juan Vivanco-Suarez, Nahom Teferi, Alex Belzer, Hend Al-Kaylani, Meron Challa, Sarah Lee, John M. Buatti, Patrick Hitchon

**Affiliations:** 1 Carver College of Medicine, University of Iowa, Iowa City, IA USA; 2 Department of Neurology, University of Iowa, Iowa City, IA USA; 3 Neurosurgery and Biomedical Engineering, Department of Neurosurgery, University of Iowa Hospitals and Clinics, 200 Hawkins Dr., Iowa City, IA 52242 USA; 4 Department of Radiation Oncology, University of Iowa, Iowa City, IA USA

**Keywords:** Malignant peripheral nerve sheath tumors, MPNST, Mesenchymal tumors, Craniospinal axis, Malignant Triton tumor

## Abstract

**Background:**

Malignant peripheral nerve sheath tumor (MPNST) is an exceedingly rare and aggressive tumor, with limited literature on its management. Herein, we present our series of surgically managed craniospinal MPNSTs, analyze their outcomes, and review the literature.

**Methods:**

We retrospectively reviewed surgically managed primary craniospinal MPNSTs treated at our institution between January 2005 and May 2023. Patient demographics, tumor features, and treatment outcomes were assessed. Neurological function was quantified using the Frankel grade and Karnofsky performance scores. Descriptive statistics, rank-sum tests, and Kaplan–Meier survival analyses were performed.

**Results:**

Eight patients satisfied the inclusion criteria (4 male, 4 female). The median age at presentation was 38 years (range 15–67). Most tumors were localized to the spine (75%), and 3 patients had neurofibromatosis type 1. The most common presenting symptoms were paresthesia (50%) and visual changes (13%). The median tumor size was 3 cm, and most tumors were oval-shaped (50%) with well-defined borders (75%). Six tumors were high grade (75%), and gross total resection was achieved in 5 patients, with subtotal resection in the remaining 3 patients. Postoperative radiotherapy and chemotherapy were performed in 6 (75%) and 4 (50%) cases, respectively. Local recurrence occurred in 5 (63%) cases, and distant metastases occurred in 2 (25%). The median overall survival was 26.7 months. Five (63%) patients died due to recurrence.

**Conclusions:**

Primary craniospinal MPNSTs are rare and have an aggressive clinical course. Early diagnosis and treatment are essential for managing these tumors. In this single-center study with a small cohort, maximal resection, low-grade pathology, young age (< 30), and adjuvant radiotherapy were associated with improved survival.

## Background

Malignant peripheral nerve sheath tumors (MPNSTs) are aggressive soft tissue sarcomas arising from peripheral nerves or associated nerve sheaths [1]. These are rare tumors, comprising 5–10% of all soft tissue sarcomas in the United States, with an overall incidence of 0.001% [2]. MPNSTs are characterized by aggressive local invasiveness and high rates of both local recurrence and distant metastases [1]. The term ‘MPNST’ was first coined by the World Health Organization (WHO) [3] in 1990; these tumors were previously referred to as ‘neurofibrosarcoma’, ‘neurogenic sarcoma’, ‘malignant neurolemmoma’, or ‘malignant schwannoma’ [4]. MPNSTs occur most frequently in patients with neurofibromatosis type 1 (NF-1). Patients with NF-1 and plexiform neurofibromas are 18 times more likely to develop MPNSTs, and 20–30% of MPNSTs occur in patients with NF-1 [1]. Radiation exposure, such as prior radiotherapy (RT), is also a risk factor for the development of MPNSTs, with 10% of MPNSTs developing in irradiated patients [1]. Primary intradural MPNSTs, however, can occur in the absence of any predisposing risk factors. MPNSTs arise most frequently in the deep soft tissues of extremities near nerve trunks; however, exact incidence rates by location are difficult to determine [5]. Primary intradural MPNSTs in the central nervous system (CNS) are exceedingly rare, with few reported cases; most are intracranial rather than spinal [6–9]. CNS MPNSTs are often misdiagnosed on imaging, with more common benign diagnoses such as meningioma or schwannoma often considered [1, 4]. When arising within the craniospinal axis, they can cause symptoms secondary to mass effect, resulting in spinal cord compression, cranial nerve palsies, and focal neurologic deficits based on tumor location [7, 10, 11]. Spinal MPNST may also invade the vertebrae and cause bony erosion [11]. MPNSTs have poor outcomes, with low progression-free survival (PFS) and overall survival (OS) [6, 12]. Outcomes are worst in craniospinal axis MPNST, with a five-year OS as low as 25%, and PFS ranging from 5 to 32.2 months [6, 9, 13]. Timely diagnosis and management of craniospinal MPNST is key to improving OS given the aggressive nature of these tumors.

The natural disease course of craniospinal MPNST, best treatment options, and associated complications are largely unknown and currently based on case reports and small case series, owing to its rarity. Additionally, no studies have rigorously described neurological or functional outcomes in surgically managed patients with craniospinal MPNSTs. To date, we present one of the larger series in the literature on MPNSTs within the craniospinal axis, analyze our treatment algorithm and patient outcomes, and extensively review the associated literature.

## Methods

### Institutional setting

The study was approved by the University of Iowa Institutional Review Board (IRB). A retrospective review of hospital records was performed for the diagnosis of ‘MPNST’, ‘malignant neoplasms of connective and soft tissue’, and ‘malignant neoplasms of spinal cord/brain’ from January 2005 to May 2023. Informed consent was waived by the IRB for all the subjects (IRB #201902751). This study was conducted at the University of Iowa Hospitals and Clinics. Chart records were obtained from the EPIC (Epic Systems Corporation, Madison, WI) electronic medical record (EMR).

### Data collection

The EMRs of two hundred twenty-four patients (224) with MPNST were initially reviewed. Eight (8) patients were identified with a diagnosis of primary intradural MPNST of the craniospinal axis. We collected information on patient demographics, clinical characteristics, radiological and pathological findings, clinical course, treatment modalities, survival, and functional outcomes. Radiological test results, including computed tomography (CT) and magnetic resonance imaging (MRI), were collected as they pertain to lesion location and appearance, involvement of craniospinal structures, and compression of neural elements. Surgical treatment modalities include craniotomy for intracranial tumor resection and laminectomy/laminoplasty for spinal tumor resection. Any distant metastasis or local tumor recurrence was noted with the corresponding mode of management, which included reoperation, salvage RT, chemotherapy (CHE), or a combination of these treatments. Neurologic status was documented using pre- and postoperative Frankel grading [14]. Karnofsky Performance Scores (KPS) [15] were collected to document functional status.

### Statistical methods

Descriptive statistics were used to describe patient demographics, tumor characteristics, clinical course, and treatment factors. Patient demographics included age, sex, and ethnicity. Tumor characteristics included tumor location, radiologic diagnosis, and WHO grade on pathologic diagnosis. Clinical course included presenting symptoms, pre- and postoperative KPS, Frankel grade, tumor recurrence, metastasis, follow-up history, and vital status as of June 2023. Treatment factors included the extent of resection (EOR), reoperation, and use of adjuvant CHE or RT.

GraphPad Prism 9 (Dotmatics LLC, San Diego, CA, USA) was used for quantitative analysis. Categorical variables were compared using Fisher’s exact test, and numerical variables were analyzed using the Mann–Whitney-Wilcoxon rank sum test. Survival analyses were performed using Kaplan–Meier estimation. OS was calculated from the date of initial surgery to the date of death reported in patient medical records. PFS was calculated from the date of initial surgery to the date of tumor recurrence found on radiological evaluation. Patients not documented as deceased or having residual tumor or tumor recurrence were censored from the date of the last follow-up for OS and PFS, respectively. The results were considered significant at a *p* value < 0.05.

## Results

### Patient demographics and clinical characteristics

A total of 8 patients with a diagnosis of craniospinal MPNST at our institution met the inclusion criteria. The clinical characteristics of all patients are summarized in Table 1. There were 4 male and 4 female patients (sex ratio 0.5), with a median age of 38 years (range 15–67 years). Most patients presented with spinal tumors (6/8, 75%). The most common presenting symptoms were paresthesia/numbness (4/8, 50%), pain (3/8, 37.5%), and weakness (2/8, 25%). Visual changes were noted in 1/2 cranial cases. The median preoperative KPS was 50 (range 30–100). Preoperative Frankel grade was most frequently D in 4 (50%) patients, followed by E in 3 (37.5%) and C in 1 (12.5%). Three (37.5%) patients had NF-1. The duration of symptoms prior to presentation ranged from 3 days to 12 months.

**Table 1:** Summary of the MPNST cases

N	Age / Sex	Clinical presentation	Symptom duration	Location	Size (cm)	EOR	Grade	Adjuvant therapy	Recurrence	Mets	KPS preop/postop	Follow-up (mo.)
1	67/M	Visual changes, CN VI palsy	4 mo	Rt orbital and superior orbital fissure/cavernous sinus	1.0	STR	High	RT	-	Lung	50/80	Alive at 69.5
2	58/M	Rt flank and LE pain, urinary retention^a^	1 mo	Rt T10-sacrum paraspinal	20.2	GTR	High	RT	1	-	30/40	Dead at 5.4
3	58/F	Lt UE numbness and weakness	12 mo	Lt C7-T1 intradural extramedullary	2.5	STR	High	CHE, RT	-	-	70/50	Dead at 37.6
4	39/M	Lt head, ear, and neck numbness and paresthesia	3 mo	Lt C2-C3 extradural extramedullary	5.2	GTR	High	CHE, RT	3	-	90/100	Dead at 22.1
5	37/F	Rt side weakness, confusion, word finding difficulty	3 d	Lt frontal lobe	1.7	STR	High	CHE, RT	3	Lung	30/100	Dead at 26.7
6	35/F	Rt lower back pain^b^	3 mo	Rt L2-L5 paraspinal (multiple other lesions non-malignant lesions present)	6.2	GTR	Low	-	-	-	100/100	Alive at 25.8
7	24/F	Rt LE paresthesia, chest pain	1 mo	Rt C7-T1 intradural extramedullary	3.0	GTR	Low	-	2	-	30/100	Alive at 105.2
8	15/M	Rt neck pain, Rt UE paresthesia^c^	5 mo	C7 extradural	2.8	STR	High	CHE, RT	1	-	50/60	Dead at 25.1

*Abbreviations*: *CHE* chemotherapy, *CN* cranial nerve, *d* days, *F* female, *GTR* gross total resection, *KPS* Karnofsky performance score, *LE* lower extremity, *Lt* left, *M* male, *Mets* metastasis, *mo* months, *RT* radiotherapy, *Rt* right, *STR* subtotal resection, *UE* upper extremity, *y* year

^a^ Patient has NF-1 + removal of 3 peripheral tumors (3 MPNSTs)

^b^ History of NF-1 + multiple superficial neurofibromas

^c^ History of NF-1 + removal of 5 peripheral tumors (3 MPNSTs and 2 plexiform neurofibromas)

### Imaging findings

**Fig. 1:**
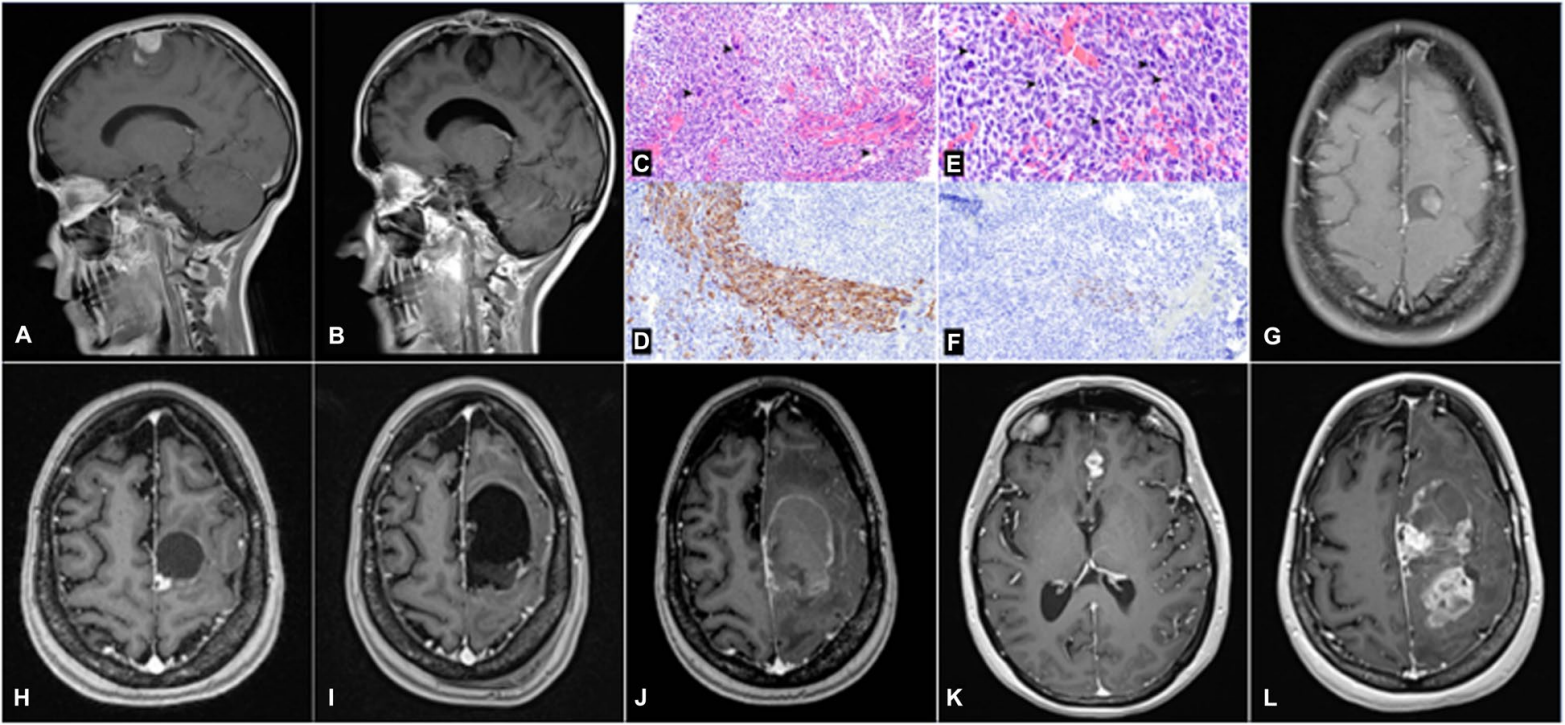
Case 2. Sagittal (**a**) and axial (**b**) post-contrast T1-weighted images showing the tumor at the right side extending from T10 to the sacrum. Post-surgical sagittal (**c**) and axial (**d**) post-contrast T1-weighted images showing the resection cavity

All patients underwent MRI of the neural axis prior to surgery. The radiological characteristics of all patients are presented in Table 2. The median tumor size was 3 cm (range 1.0–20.2 cm). The tumors were oval in 4 (50%) cases and dumbbell or irregular in 2 cases each (25%). Most tumors had well-defined borders (6/8, 75%). Two (25%) tumors were located intracranially. Of the spinal tumors, 2 (33.3%) were cervical, 2 (33.3%) cervicothoracic, 1 (16.7%) lumbar, and 1 (16.7%) thoracolumbosacral (Fig. 1). On T1-weighted imaging, 4 (50%) lesions were hypointense, 3 (37.5%) were isointense, and 1 (12.5%) was hyperintense, while on T2-weighted imaging, 4 (50%) tumors were hyperintense, 3 (37.5%) were isointense, and 1 (12%) had heterogeneous intensity. Only 1 (12.5%) tumor did not show contrast enhancement, and 1 (12.5%) tumor had intramedullary extension. In 3 (37.5%) cases, peripheral tumors were diagnosed and excised before MPNST diagnosis. Based on imaging findings, 5 patients with primary intradural MPNSTs in our study were preoperatively misdiagnosed as having other lesions, including schwannoma (3 cases), meningioma (1 case), and neurofibroma (1 case).

**Table 2:** Radiological characteristics of the MPNST cases

Characteristic	No. of cases
**Size**	
≤ 3 cm	4
> 3 cm	4
**Shape**	
Oval	4
Dumbbell	2
Irregular	2
**Border of the tumor**	
Well defined	6
Poorly defined	2
**Magnetic resonance imaging findings**	
**T1-weighted sequence**	
Hyperintense	1
Isointense	3
Hypointense	4
**T2-weighted sequence**	
Hyperintense	4
Isointense	3
Heterogenous	1
**Contrast enhancement**	
Yes	7
No	1
**Bone erosion**	
Yes	2
No	6

### Pathology findings

**Fig. 2:**
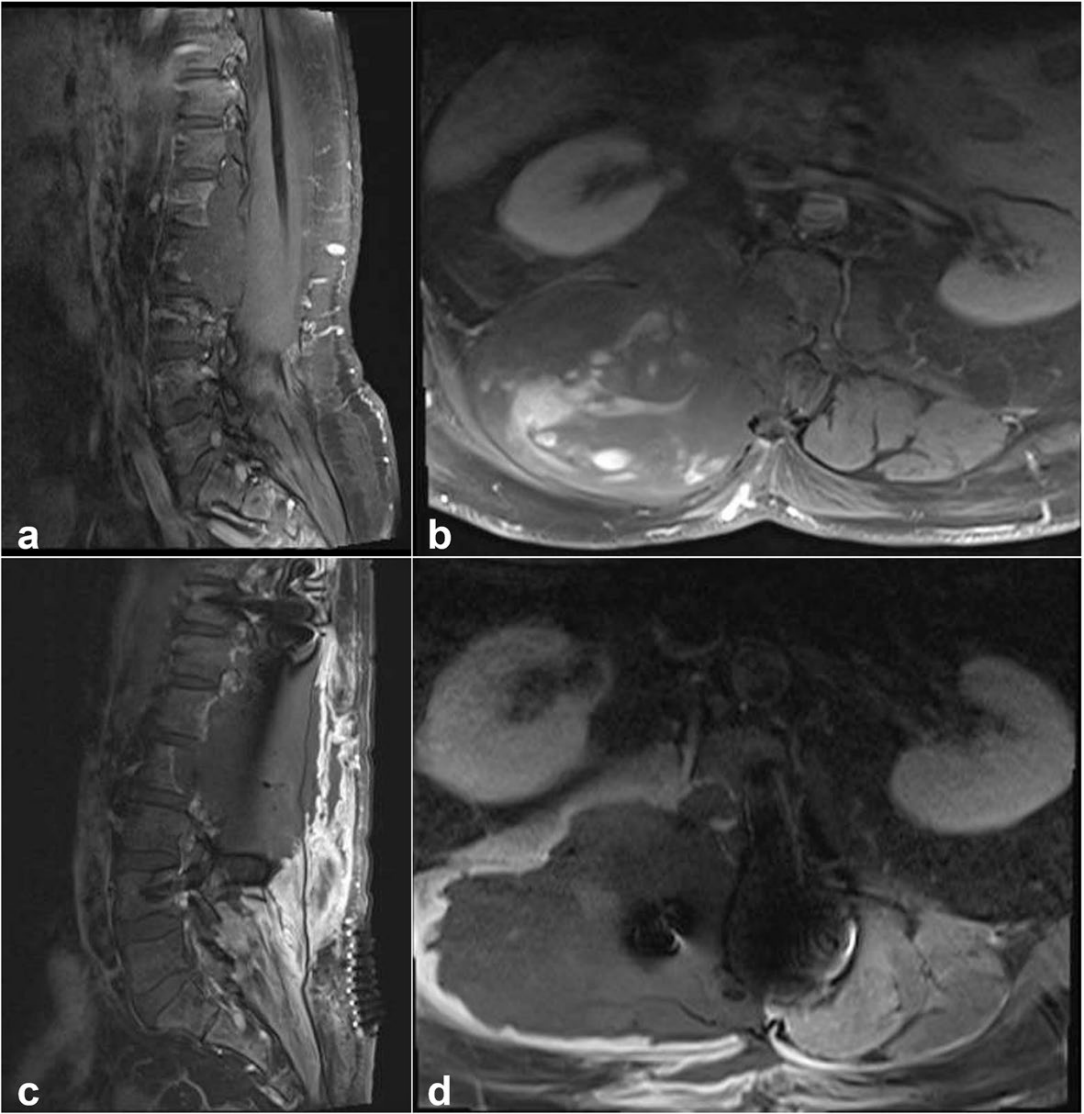
Case 5. Sagittal (**a**) and axial (**b**) post-contrast T1-weighted images showing the tumor at the left frontal lobe. Post-surgical sagittal (**c**) and axial (**d**) post-contrast T1-weighted images showing the resection cavity. Tumor pathologic sample (**e**) [hematoxylin–eosin 200 × magnification] showing hypercellularity, fascicles of hyperchromatic spindled cells with pale cytoplasm, and scattered rhabdomyoblastic cells (arrowheads). Immunohistochemical stains for desmin (**f**) and myogenin (**g**). Axial (**h**) post-contrast T1-weighted images showing final tumor progression before the patient passed

On histological hematoxylin–eosin (H&E) examination, the tumors typically presented with spindle-shaped morphology with pleomorphic, hyperchromatic, and atypical nuclei arranged in a fascicular architecture. One patient presented with malignant Triton tumor (MTT) histology (Fig. 2), a high-grade MPNST with focal rhabdomyoblastic differentiation, focal expression of desmin and myogenin, and loss of H3K27me3 expression (Fig. 2e-g). On immunohistochemistry (IHC), S100 was positive in 5 (62.5%) cases, vimentin in 4 (50%), SMA in 2 (25%), desmin in 1 (12.5%), and EMA in 1 (12.5%). According to the WHO classification, 6 (75%) tumors were high grade, and 2 (25%) were low grade [3].

### Management outcomes

The nerve from which the tumor originated was identified in 6 (75%) cases. Gross total resection (GTR) was achieved in 5 (62.5%) cases, and subtotal resection (STR) was performed in the remaining 3 (37.5%). Adjuvant RT after surgery was performed in 6 (75%) patients with a median dose of 60 Gy (range 30–72 Gy). Two patients (25%) had GTR and low-grade tumors on histology and did not receive adjuvant RT. CHE was administered in 4 (50%) cases. Local recurrence was observed in 5 (62.5%) cases, and in 2 (25%) cases, patients presented with distant metastases to the lung. One patient experienced both local recurrence and distant metastasis (1/8, 12.5%). Five out of six (83.3%) patients with local recurrence or distant metastasis received adjuvant RT postoperatively, and half received adjuvant CHE (3/6, 50%). Three patients (3/5, 60%) with local recurrence underwent reoperation.

**Fig. 3:**
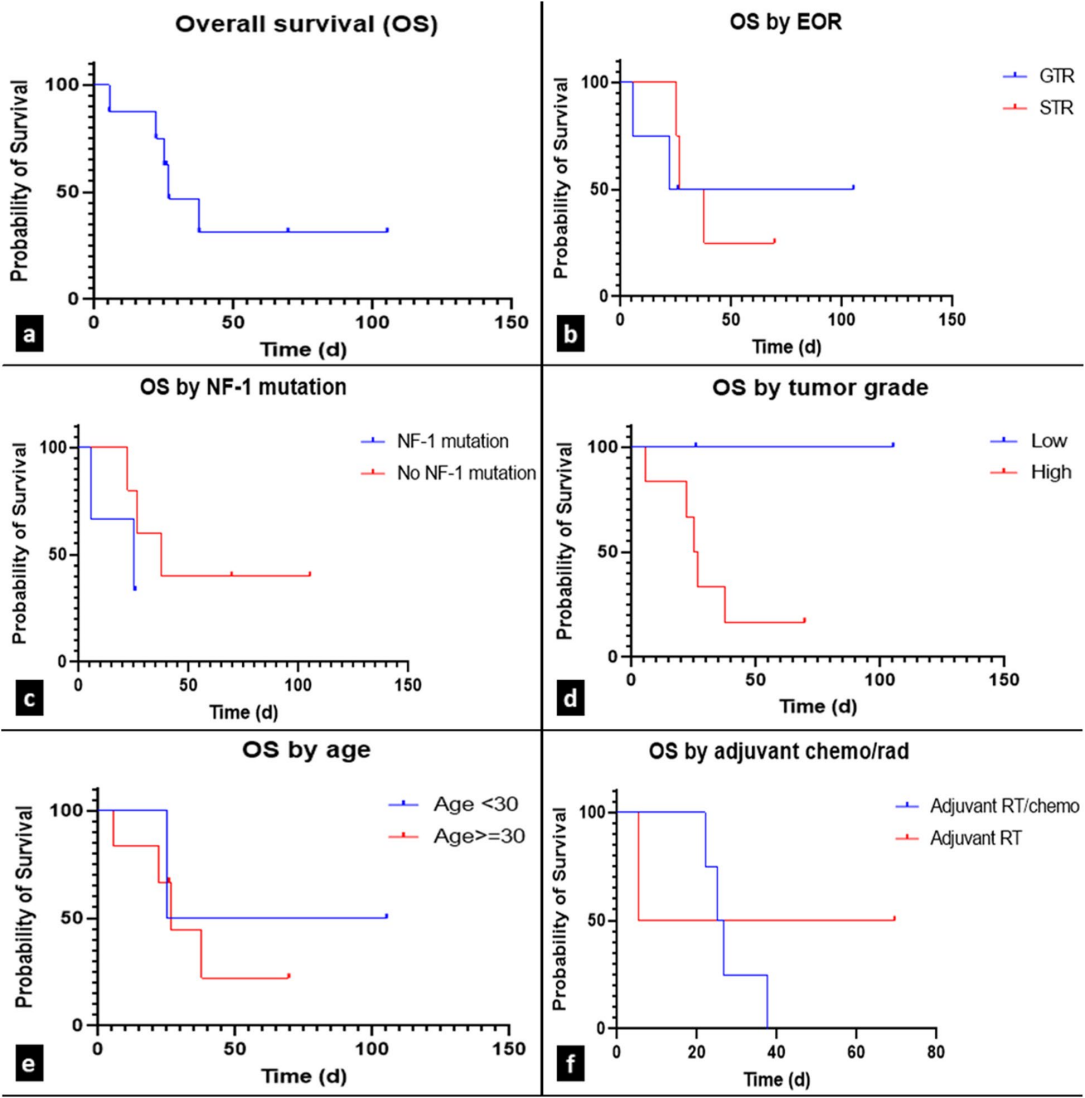
Kaplan–Meier survival curves. Overall survival (**a**) in patients with a diagnosed MPNST. Survival by the following: extent of resection (**b**), presence of NF-1 mutation (**c**), tumor grade (d), age (e) and adjuvant chemotherapy/radiation (**f**). Abbreviations: extent of resection (EOR); gross total resection (GTR); neurofibromatosis type 1 (NF-1); radiotherapy (RT); subtotal resection (STR)

The median postoperative KPS was 90 (range 40–100). The postoperative Frankel grade was E in 5 (63%) patients, D in 2 (25%) and C in 1 (12%). The median PFS was 19.5 months, and the median OS was 26.7 months (Fig. 3a). When stratified by EOR, the median OS was 63.7 months (range 5.4–105.2 months) in patients with GTR and 32.15 months (range 25.1–69.5 months) in patients with STR (Fig. 3b). Similarly, patients without NF-1 mutations, low-grade tumors, and younger age (< 30 years) (median OS 37.6 months; not reached, 65.2 months) were found to have a longer OS than patients with NF-1 mutations, high-grade pathology and older age (age > 30) (median OS 25.1 months, 25.9 months, 26.7 months, respectively) (Fig. 3c-e). Patients who received adjuvant chemoradiation had lower OS (25.9 months) than patients who received adjuvant RT alone (37.5 months) (Fig. 3f), likely due to poor prognostic features of advanced disease/metastases. Given the small sample size in each cohort, these survival observations trended toward significance but were not statistically significant (*p* < 0.5). Death had occurred in 5 (62.5%) cases at the time of data collection. The most common causes of death were local disease recurrence and increased tumor burden, all attributed to MPNST.

### Complications

One patient developed cephalic vein thrombosis from their intravenous line, which resolved spontaneously after line removal. One patient developed pneumonia postoperatively, which was managed with antibiotics and resolved after 7 days. No complications were directly attributed to surgical intervention.

## Discussion

MPNSTs are highly recurrent, aggressive soft tissue sarcomas with a tendency to metastasize [6, 9, 13] and have an incidence of approximately 0.001% in the general population [2]. They are thought to arise from peripheral nerves or their associated nerve sheaths [1]; however, Rubino et al. hypothesized that they originate from the nervi vasorum, which are autonomic peripheral nerves in the adventitial layer of the large and small pial arteries [16]. Primary MPNSTs of CNS origin are even less common and are analogous to the malignant version of schwannomas [16]. A history of NF-1 or prior irradiation are important risk factors in the development of MPNST [1]; however, they do not necessarily compose most cases of MPNST, with only 20–30% of patients having NF-1 and only 10% of patients reporting prior radiation exposure [1]. Sex is not a known risk factor for this tumor [1]. Many of these epidemiological characteristics of MPNST are reflected in our cohort; it was demographically evenly split between males and females, and 3 (37.5%) of our patients had NF-1. Interestingly, one patient had a history of RT for Hodgkin’s lymphoma and was diagnosed with MTT, a specific subtype of MPNST with an even worse prognosis [17].

Craniospinal axis MPNSTs are exceedingly rare, with approximately 100 cases of intracranial tumors [7, 18, 19] and dozens of spinal tumors [6, 8, 13, 20–22] reported in the literature, indicating that intracranial location may be more common than spinal location. Our cohort’s composition of craniospinal MPNST locations deviates from this, with 75% of patients having spinal tumors.

Patients with primary intradural MPNST often present with insidious neurological symptoms, which are generally attributable to a progressive mass effect on nearby neurovascular structures. When present in the cranium, symptoms include headache, nausea/vomiting, seizures, focal neurological deficits, and/or cranial nerve palsies [1]. When present in the spine, MPNST may cause myelopathic symptoms, pain, motor weakness, sensory deficit/radiculopathy, and bowel/bladder dysfunction [1]. These symptoms were observed in our cohort (Table 1).

### Clinical diagnostic workup, radiologic findings, and histopathology

Timely and accurate diagnosis of MPNST is difficult and often requires extensive workup. A thorough history and physical examination, noting the onset and duration of symptoms, are necessary, as rapid progression of symptoms would be concerning for malignancy. It is also important to elicit a past medical or family history of NF-1, schwannomatosis, or prior RT. On physical examination, the typical findings of NF-1, such as café-au-lait spots, Lisch nodules, and cutaneous neurofibromas, should be evaluated. Diligent neurological evaluation of sensory, motor, and gait functions is key to localizing lesions in the craniospinal axis.

Following a detailed history and physical examination, further workup primarily consists of imaging with CT and MRI. Contrast-enhanced MRI with gadolinium has the highest resolution and is considered the imaging modality of choice [23]. Radiologic differential diagnoses often considered for MPNSTs include meningiomas, solitary fibrous tumors/hemangiopericytomas, schwannomas, and dural-based metastases. These were considered in radiological reports of our cohort. Certain radiologic features favor a diagnosis of MPNST over benign lesions such as neurofibroma or schwannoma, including size > 5 cm, ill-defined borders, soft tissue edema, lobulation, lack of a target sign, and surrounding bone destruction [23]. The radiological findings of our patient cohort are outlined in Table 2.

On histopathological examination, MPNSTs are usually high-grade malignant spindle cell tumors most commonly found in nerves. More specifically, they arise from preexisting Schwann cell tumors (such as plexiform neurofibromas) [24], and H&E microscopy typically reveals a cellular neoplasm with fascicles comprising spindly cells with tapered hyperchromatic nuclei [25]. Mitotic figures and necrosis are common [26] but are notably decreased in low-grade tumors [24]. A marble-like appearance may be seen at low power, with further evaluation under high power revealing alternating hyper and hypocellular areas [24]. MPNSTs also have incredible plasticity and may demonstrate internal cartilage, bone, skeletal or smooth muscle, glandular, epithelioid, and/or perineural differentiation [24, 25, 27]. Macroscopically, these tumors are highly variable in size and are adherent and exophytic with common areas of hemorrhage and necrosis. Our cohort showed many of these histopathological findings. Further analysis using IHC is routinely performed, but given heterogeneous findings, no standard set of diagnostic characteristics exists. The highest yields are for the S100 and SOX10 stains, which are often decreased relative to other neural crest-originating tumors and are often correlated [26, 28]. S100 may be particularly useful for distinguishing MPNST from malignant melanoma [29]. This deviates from our cohort’s IHC analysis, as 5 (63%) of our patients had a positive S100. The loss of p16 is also typical [26]. Other common traits include loss of neurofibromin expression (which is more common in NF-1-associated than sporadic tumors) [26] and H3K27 trimethylation. Loss of the latter is highly specific for MPNST and is associated with worse survival [30].

### Management and outcomes

Currently, there is no standard therapeutic approach for MPNSTs. Maximal safe gross total resection with negative margins is recommended when feasible but is often difficult to achieve, as these tumors tend to grow near vital neurovascular structures [1, 4, 19]. Our cohort underwent extensive and variable treatment regimens with multidisciplinary teams involving neurosurgery and oncology, with patients receiving a combination of RT, CHE, or observation postoperatively. The general approach of maximal surgical resection to improve survival was true in our cohort, in which patients who underwent GTR survived longer than those treated with STR (Fig. 3b). Our findings strengthen this approach, and we recommend maximal safe surgical resection for the surgical management of MPNST.

There is little available literature on the effect of surgical treatment on neurological and functional outcomes in craniospinal MPNST. Our study found that 87.5% of patients had an improvement in their postoperative functional status as quantified by KPS; however, a larger sample size is warranted prior to making definitive conclusions.

Because of the radiation-inducible nature of MPNSTs and increased radiation sensitivity of patients with NF-1, adjuvant RT has not been shown to improve OS for MPNST, with many studies finding that RT may improve local control of disease and lengthen PFS but does not improve OS [1, 4, 6, 19, 31, 32]. Despite this, RT has been shown to improve OS in the management of MTT, as in Case 5 in our cohort [33]. All 6 of the patients in our cohort with high-grade tumor pathology received RT with subsequent improved OS compared to historical cohorts; however, due to the small number of patients in this study, the comparative analysis did not reach significance. MPNST has historically been shown to be poorly responsive to CHE [31, 32], and it is notable that in our cohort, despite not reaching significance, patients treated with adjuvant RT alone had higher survival rates (OS 37.5 months) than patients treated with chemoradiation (25.9 months) (Fig. 3d). We recommend adjuvant RT for the management of MPNST; however, further multicenter reviews and randomized clinical trials are necessary to further strengthen this recommendation.

Survival in MPNST is very poor, with 5-year survival rates as low as 25% owing to increased local recurrence and metastasis rates [6, 12]. Local recurrence rates reported range from 31 to 75%, with a median PFS of 5 to 32.2 months [6, 9, 13] and distal metastasis rates of 22% to 45% [9, 13]. Negative prognostic factors reported include tumor size over 5 cm, higher tumor grade, positive surgical margin, positive NF-1 status, and Ki-67 score over 20. This was further corroborated in our cohort with patients undergoing GTR of tumor, low grade tumor pathology on histology, and absence of NF-1 mutation trending toward having an improved OS (Fig. 3b, c) [1, 4, 9].

### Limitations

Given the rarity of this pathology and the small number of patients expected from a single-center study, we extensively reviewed the literature to further strengthen our recommendations. The retrospective and nonrandomized nature of this study also decreased the level of evidence. Controlled multicenter large-scale studies are necessary to recommend stronger guidelines.

## Conclusions

The highly aggressive, recurrent, and metastatic characteristics of primary craniospinal axis MPNST, along with its rarity, pose many challenges. Radiation exposure and positive NF-1 status both increase the risk of developing MPNST and worsen prognosis. Initial presenting symptoms are secondary to the mass effect on nearby neural structures. Gadolinium contrast-enhanced MRI is recommended for imaging, along with histopathological analysis, to confirm diagnosis. Maximal tumor resection has consistently been shown to improve survival in patients with MPNST, and RT shows promise as an adjuvant treatment. To date, nonsurgical management of MPNST has not been found to improve outcomes. A multidisciplinary team of neurosurgeons, radiologists, pathologists, and oncologists is essential to optimally diagnose and manage MPNST.

## Data Availability

The datasets used and/or analysed during the current study are available from the corresponding author on reasonable request.

## Abbreviations

MPNST: Malignant peripheral nerve sheath tumors
WHO: World Health Organization
NF-1: Neurofibromatosis type 1
RT: Radiotherapy
CNS: Central nervous system
PFS: Progression-free survival
OS: Overall survival
IRB: Institutional Review Board
EMR: Electronic medical record
CT: Computed tomography
MRI: Magnetic resonance imaging
CHE: Chemotherapy
KPS: Karnofsky Performance Scores
EOR: Extent of resection
H&E: Hematoxylin-eosin
MTT: Malignant Triton tumor
IHC: Immunohistochemistry
GTR: Gross total resection
STR: Subtotal resection
